# Nonlinear Encryption for Multiple Images Based on a Joint Transform Correlator and the Gyrator Transform

**DOI:** 10.3390/s23031679

**Published:** 2023-02-03

**Authors:** Ronal A. Perez, Juan M. Vilardy, Elisabet Pérez-Cabré, María S. Millán, Cesar O. Torres

**Affiliations:** 1 Grupo de Investigación en Física del Estado Sólido (GIFES), Faculty of Basic and Applied Sciences, Universidad de La Guajira, Riohacha 440007, La Guajira, Colombia; 2 Applied Optics and Image Processing Group, Universitat Politècnica de Catalunya · BarcelonaTech, 08222 Terrassa, Barcelona, Spain; 3 Grupo de Óptica e Informática, Department of Physics, Universidad Popular del Cesar, Valledupar 200001, Cesar, Colombia

**Keywords:** optical multiple-image encryption–decryption system, joint transform correlator (JTC), Gyrator transform, multispectral images, nonlinear image processing

## Abstract

A novel nonlinear encryption–decryption system based on a joint transform correlator (JTC) and the Gyrator transform (GT) for the simultaneous encryption and decryption of multiple images in grayscale is proposed. This security system features a high level of security for the single real-valued encrypted image and a high image quality for the multiple decrypted images. The multispectral or color images are considered as a special case, taking each color component as a grayscale image. All multiple grayscale images (original images) to encrypt are encoded in phase and placed in the input plane of the JTC at the same time without overlapping. We introduce two random-phase masks (RPMs) keys for each image to encrypt at the input plane of the JTC-based encryption system. The total number of the RPM keys is given by the double of the total number of the grayscale images to be encrypted. The use of several RPMs as keys improves the security of the encrypted image. The joint Gyrator power distribution (JGPD) is the intensity of the GT of the input plane of the JTC. We obtain only a single real-valued encrypted image with a high level of security for all the multiple grayscale images to encrypt by introducing two new suitable nonlinear modifications on the JGPD. The security keys are given by the RPMs and the rotation angle of the GT. The decryption system is implemented by two successive GTs applied to the encrypted image and the security keys given by the RPMs and considering the rotation angle of the GT. We can simultaneously retrieve the various information of the original images at the output plane of the decryption system when all the security keys are correct. Another result due to the appropriate definition of the two nonlinear operations applied on the JGPD is the retrieval of the multiple decrypted images with a high image quality. The numerical simulations are computed with the purpose of demonstrating the validity and performance of the novel encryption–decryption system.

## 1. Introduction

The research field of optical image encryption has shown an intense development due to the advantages provided by the optical processing systems, such as a high parallel processing capacity, an ultrafast computing speed and the wide variety of controllable physical parameters of these optical systems, and also because of the several applications in optical security published in the last years [1,2,3,4,5,6,7]. The most known optical technique for image encryption is the double random-phase encoding (DRPE) proposed by Réfrégier and Javidi [8]. The DRPE can be optically implemented using a classical 4*f*-processor [9] or a joint transform correlator (JTC) [10]. The image to encrypt by the optical DRPE is converted into a stationary white noise image (encrypted image) by using two random-phase masks (RPMs). Initially, the DRPE was proposed in the Fourier domain using the 4*f*-processor. Later, the DRPE was extended from the Fourier domain to the Fresnel domain [11], the fractional Fourier domain (FrFD) [12] and the Collins diffraction domain [13], with the purpose of increasing the security of the DRPE technique.

Nowadays, the DRPE is implemented by using a JTC architecture [1,4,5,6,7,10,14,15,16,17] due to the following advantages: the compact size of the experimental setup, a less strict setup alignment requirement, the encrypted image is a real-valued distribution and the security key used in the encryption system is exactly the same to be used in the decryption system. The first implementations of the DRPE using a JTC architecture were proposed in the Fourier domain. The DRPE implemented with a JTC architecture was also extended from the Fourier domain to the Fresnel domain [18,19,20], the FrFD [21,22,23,24], the Gyrator domain (GD) [25,26] and the Collins diffraction domain [27] in order to either simplify the experimental setup and/or to increase the security of the encryption system.

The security of the optical architectures that implement the DRPE based on a 4*f*-processor were analyzed and it was shown that these optical implementations are vulnerable to chosen-plaintext attacks (CPA) [28,29], known-plaintext attacks (KPA) [29,30] and ciphertext-only attacks (COA) [31]. The linear property of the 4*f*-processor is the main reason for these vulnerabilities of the DRPE technique [29]. Some papers have proved that the optical DRPE implemented by using a JTC architecture is also vulnerable to CPA [32], KPA [33,34] and COA [35]. The initial JTC architecture used to implement the DRPE was nonlinear modified and extended to several optical processing domains in order to improve the security of the encryption system and the quality of the decrypted image [15,16,17,18,19,20,21,22,23,24,25,26,27]. The encryption–decryption system based on a nonlinear JTC in the GD has shown a higher sensitivity for the rotation angle of the GT with respect to the resulting decrypted images in comparison to the sensitivity for the fractional order of the encryption–decryption system based on a nonlinear JTC in the FrFD [26]. The rotation angle of the GT can be considered as a security key of the encryption system. Therefore, the nonlinear JTC-based encryption system in the GD can be considered more secure with respect to other encryption systems that use other optical processing domains.

The optical DRPE was originally designed to encrypt either a single binary or grayscale image [8]. Later, the DRPE based on the 4*f*-processor was used to perform color image encryption [36,37]. Some implementations to encrypt color images by using the DRPE on a JTC were presented in [21,38]. The multiple-image encryption (for instance, various frames of a video sequence) by using an optical JTC architecture was also presented in some contributions [39,40]. These optical color image and multiple-image encryption systems use different input illumination wavelengths and several key codes or RPMs that can be utilized as new security keys to improve the security level of the encryption system. Optical encryption methods recently included the ability to cipher multiple images [41,42,43,44,45,46,47]. In general, this approach achieves a higher level of security because two or more pieces of information are encoded in the encryption procedure, and their decryption allows for the validation of more than one signal, unlike the first systems that dealt with a unique primary image. Different techniques were explored to achieve a successful multiple-image optical encryption and decryption. For instance, reference [41] presents a multiple-image encryption system based on a linear JTC in the Fourier domain. In [42], a modified iterative phase retrieval algorithm with a structured phase mask was used in the Fresnel domain to encrypt multiple images. Reference [43] implements a multiple-image encryption method by using phase jump gradient factors based on orbital angular momentum and multiplexing holography. Recently, a multiple-image compression, encryption and reconstruction scheme based on deep learning-assisted single-pixel imaging and orthogonal coding was presented in [44]. A sequential multiple-image encryption based on quick response (QR) codes and a modified DRPE in the FrFD was developed in [45], where each image to encrypt was converted into a QR and then this converted image was encrypted. The multiple-image encryption system in [46] was based on the optical interference by wavelength multiplexing in the Fresnel domain in order to generate two encrypted images. A nonlinear multiple-image encryption method was described in [47] by using optical scanning holography with a random-phase mask (RPM) and orthogonal compressive sensing.

This paper aims to propose a novel approach to multiple-image encryption with several differences with respect to the previously reported works. We propose a nonlinear JTC-based encryption system in the GD to encrypt multiple images in grayscale. The color or multispectral images are considered as a special case, taking each color or spectral component as a grayscale image. We present a modification of the nonlinear JTC-based encryption system proposed in [26] with the purpose of achieving the simultaneous encryption of multiple grayscale images. All the multiple grayscale images to encrypt are encoded in phase and placed in the input plane of the JTC without overlapping. We introduce a new RPM key for each image to encrypt. The total number of the RPM keys is given by the double of the total number of the grayscale images to be encrypted. The use of several RPMs as keys improves the security of the encrypted image. The joint Gyrator power distribution (JGPD) is the intensity of the Gyrator transform (GT) of the input plane of the JTC. The single real-valued encrypted image is computed by using two new nonlinear terms applied to the JGPD. The proposed multiple-image encryption system based on a nonlinear JTC in the GD retains the following advantages of the security system proposed in [26]: the improved quality of the decrypted images; shift-invariance property with respect to the lateral displacements of the RPM key in the decryption process and the retrieval of the primary images; an additional key given by the value of the rotation angle of the GT; the use of a simplified JTC in the GD that avoids the beam splitting required by other optical JTC implementations; and there is not a significant increase in the amount of information to be transmitted because the resulting encrypted function has the same size as its original version.

The proposed encryption technique allows a fast encryption time in comparison to previous proposals that sequentially encrypt multiple grayscale images [39,40]. This is because we simultaneously encrypt all the primary images, and the optical schematic of the encryption system is performed thrice in order to obtain only a single encrypted image with the hidden information of the whole set of images to encrypt (in the following example, it is up to eight signals). The multiple grayscale images encryption system of this work shows an improved security over the encrypted distribution because the nonlinear modifications of the JTC architecture in the GD depend on the number and the values of the RPM keys utilized for the encryption process. Finally, the proposed security system allows a simultaneous encryption and decryption of multiple images with a high level of security for the single real-valued encrypted image and a retrieval with a high image quality for the multiple decrypted images, due to the phase encoding of the multiple images to encrypt and the two new nonlinear operations applied on the JGPD. We point out that these two nonlinear terms are specially designed for a satisfactory multiple-image encryption and decryption, and they differ from the nonlinear modifications presented in [26].

## 2. Multiple-Image Encryption System Based on a Nonlinear JTC Architecture and the Gyrator Transform

### 2.1. Encryption Scheme

In this section, we describe the encryption scheme using the equations of a nonlinear JTC architecture in the GD [26], with the purpose of encrypting *p* grayscale images. Each grayscale image to be encrypted is denoted by a real-valued function $f_{j}\left(x,y\right)$ with values in the interval [0, 1] and j=1,2,3,…,p; each grayscale image is encoded in phase

(1) $$\begin{array}{c} f_{j,Ph}\left(x,y\right)=\exp\left\{i2\pi f_{j}\left(x,y\right)\right\}. \end{array}$$

We use two different RPMs, $r_{j}\left(x,y\right)$ and $h_{j}\left(x,y\right)$, for each grayscale image to encrypt. These two RPMs are defined by

(2) $$\begin{array}{c} r_{j}\left(x,y\right)=\exp\left\{i2\pi s_{j}\left(x,y\right)\right\},\;h_{j}\left(x,y\right)=\exp\left\{i2\pi n_{j}\left(x,y\right)\right\}, \end{array}$$

where $s_{j}\left(x,y\right)$ and $n_{j}\left(x,y\right)$ are normalized positive functions randomly generated, statistically independent and uniformly distributed in the interval [0, 1]. The functions $f_{j}\left(x,y\right)$, $s_{j}\left(x,y\right)$ and $n_{j}\left(x,y\right)$ are grayscale images with M×N pixel size. We define the new function $g_{j}\left(x,y\right)=f_{j,Ph}\left(x,y\right)r_{j}\left(x,y\right)$ given by a grayscale image encoded in phase $f_{j,Ph}\left(x,y\right)$ bonded to an RPM $r_{j}\left(x,y\right)$, with the purpose of simplifying the following equations.

**Figure 1:**
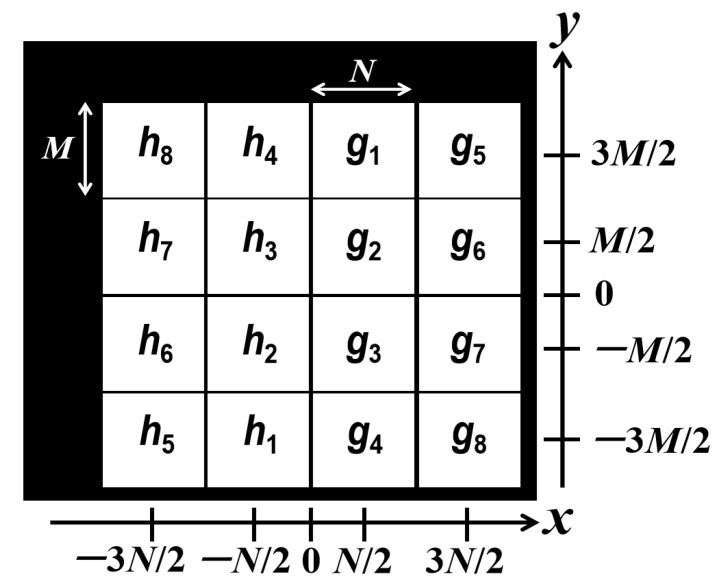
Data distributions placed at the input plane of the JTC for the p=8 grayscale images to be encrypted. The values of $a_{j}$ and $b_{j}$ with j=1,2,…,8 for this figure are $a_{1}=a_{2}=a_{3}=a_{4}=N/2$, $a_{5}=a_{6}=a_{7}=a_{8}=3N/2$, $b_{1}=b_{5}=3M/2$, $b_{2}=b_{6}=M/2$, $b_{3}=b_{7}=-M/2$ and $b_{4}=b_{8}=-3M/2$.

The input plane of the JTC is composed of two non-overlapping data distributions for each grayscale image to be encrypted. These two data distributions are the new function $g_{j}\left(x,y\right)$ and the RPM $h_{j}\left(x,y\right)$ placed anti-symmetrically side by side at the input plane of the JTC by means of the generalized shift operators ${\mathrm{GS}}_{a_{j},b_{j};\alpha }$ and ${\mathrm{GS}}_{-a_{j},-b_{j};\alpha }$, respectively, where $a_{j}$ and $b_{j}$ are real values and α is the rotation angle of the GT operator. The operators of GT and generalized shift are described in Appendix A and Appendix B, respectively. The values of $a_{j}$ and $b_{j}$ are the central points of each data distribution $g_{j}\left(x,y\right)$ and we define $a_{j}$ and $b_{j}$ proportionally to N/2 and M/2 depending on the location of each function $g_{j}\left(x,y\right)$. The distributions contained in the input plane of the JTC for all the grayscale images to be encrypted are depicted in Figure 1. The GT at parameter α of the distributions $g_{j}\left(x,y\right)$ and $h_{j}\left(x,y\right)$ are denoted by $g_{j,\alpha }\left(u,v\right)=G^{\alpha }\left\{g_{j}\left(x,y\right)\right\}$ and $h_{j,\alpha }\left(u,v\right)=G^{\alpha }\left\{h_{j}\left(x,y\right)\right\}$, respectively.

Therefore, the input plane of the JTC-based encryption scheme for all the *p* grayscale images to be encrypted is

(3) $$\begin{array}{cc} t(x,y)= & \sum_{j=1}^{p}\left\{{\mathrm{GS}}_{a_{j},b_{j};\alpha }\left[g_{j}\left(x,y\right)\right]+{\mathrm{GS}}_{-a_{j},-b_{j};\alpha }\left[h_{j}\left(x,y\right)\right]\right\}\\ = & \sum_{j=1}^{p}\{\exp\left\{-i2\pi \left[b_{j}\left(x-\frac{a_{j}}{2}\right)+a_{j}\left(y-\frac{b_{j}}{2}\right)\right]\cot\alpha \right\}g_{j}\left(x-a_{j},y-b_{j}\right)\\ & +\exp\left\{i2\pi \left[b_{j}\left(x+\frac{a_{j}}{2}\right)+a_{j}\left(y+\frac{b_{j}}{2}\right)\right]\cot\alpha \right\}h_{j}\left(x+a_{j},y+b_{j}\right)\}. \end{array}$$

Equation (3) is mathematically compact due to the chosen location of each data distribution in the input plane of the JTC (Figure 1). Other ways of placing the data distributions at the input plane of the JTC would be possible, but the resulting mathematical expression of Equation (3) would be longer and more complicated. The JGPD at parameter α for Equation (3) is the intensity of the GT of the input plane of the JTC given by [26]

(4) $$\begin{array}{cc} \mathrm{JGPD}{}_{\alpha }\left(u,v\right)= & {\left|G^{\alpha }\left\{t\left(x,y\right)\right\}\right|}^{2}={\left|\sum_{j=1}^{p}G^{\alpha }\left\{{\mathrm{GS}}_{a_{j},b_{j};\alpha }\left[g_{j}\left(x,y\right)\right]+{\mathrm{GS}}_{-a_{j},-b_{j};\alpha }\left[h_{j}\left(x,y\right)\right]\right\}\right|}^{2}\\ = & \left[\sum_{j=1}^{p}\left\{e^{-T_{j}}g_{j,\alpha }\left(u,v\right)+e^{T_{j}}h_{j,\alpha }\left(u,v\right)\right\}\right]\left[\sum_{k=1}^{p}\left\{e^{T_{k}}g_{k,\alpha }^{*}\left(u,v\right)+e^{-T_{k}}h_{k,\alpha }^{*}\left(u,v\right)\right\}\right]\\ = & \sum_{j=1}^{p}\sum_{k=1}^{p}\{e^{-T_{j}+T_{k}}g_{j,\alpha }\left(u,v\right)g_{k,\alpha }^{*}\left(u,v\right)+e^{T_{j}-T_{k}}h_{j,\alpha }\left(u,v\right)h_{k,\alpha }^{*}\left(u,v\right)\\ & +e^{T_{j}+T_{k}}g_{k,\alpha }^{*}\left(u,v\right)h_{j,\alpha }\left(u,v\right)+e^{-T_{j}-T_{k}}g_{j,\alpha }\left(u,v\right)h_{k,\alpha }^{*}\left(u,v\right)\}, \end{array}$$

where the variables *u* and *v* are the output coordinates in the GD, $T_{j}=i2\pi \left(b_{j}u+a_{j}v\right)\csc\alpha$, $T_{k}=i2\pi \left(b_{k}u+a_{k}v\right)\csc\alpha$ and the superscript ${}^{*}$ denotes the complex conjugation operation. The JGPD is a positive real-valued distribution and it has $4p^{2}$, being *p* the total number of original images to encrypt. The four general terms of the double summation in Equation (4) are composed of the multiplication of different pure linear phase terms and the products between the data distributions $g_{j,\alpha }\left(u,v\right)$ and $g_{k,\alpha }^{*}\left(u,v\right)$, $h_{j,\alpha }\left(u,v\right)$ and $h_{k,\alpha }^{*}\left(u,v\right)$, $g_{k,\alpha }^{*}\left(u,v\right)$ and $h_{j,\alpha }\left(u,v\right)$, and $g_{j,\alpha }\left(u,v\right)$ and $h_{k,\alpha }^{*}\left(u,v\right)$, respectively. We define the intensities $I_{1}\left(u,v\right)$ and $I_{2}\left(u,v\right)$ as

(5) $$\begin{array}{cc} I_{1}\left(u,v\right)= & {\left|\sum_{j=1}^{p}G^{\alpha }\left\{{\mathrm{GS}}_{a_{j},b_{j};\alpha }\left[g_{j}\left(x,y\right)\right]\right\}\right|}^{2}=\sum_{j=1}^{p}\sum_{k=1}^{p}e^{-T_{j}+T_{k}}g_{j,\alpha }\left(u,v\right)g_{k,\alpha }^{*}\left(u,v\right),\\ I_{2}\left(u,v\right)= & {\left|\sum_{j=1}^{p}G^{\alpha }\left\{{\mathrm{GS}}_{-a_{j},-b_{j};\alpha }\left[h_{j}\left(x,y\right)\right]\right\}\right|}^{2}=\sum_{j=1}^{p}\sum_{k=1}^{p}e^{T_{j}-T_{k}}h_{j,\alpha }\left(u,v\right)h_{k,\alpha }^{*}\left(u,v\right). \end{array}$$

The specific location of each data distribution depicted in Figure 1 for the input plane of the JTC allows an easy computation of the intensities $I_{1}\left(u,v\right)$ and $I_{2}\left(u,v\right)$ defined in Equation (5) by using a GT implemented optically or numerically. The next step in the encryption scheme is to subtract the intensities $I_{1}\left(u,v\right)$ and $I_{2}\left(u,v\right)$ from the JGPD. Then, the previous modification of the JGPD is divided by the intensity $I_{2}\left(u,v\right)$ with the purpose of obtaining the encrypted image

(6) $$\begin{array}{cc} e{}_{\alpha }\left(u,v\right)= & \frac{\mathrm{JGPD}{}_{\alpha }\left(u,v\right)-I_{1}\left(u,v\right)-I_{2}\left(u,v\right)}{I_{2}\left(u,v\right)}\\ = & \frac{1}{I_{2}\left(u,v\right)}\sum_{j=1}^{p}\sum_{k=1}^{p}\left\{e^{T_{j}+T_{k}}g_{k,\alpha }^{*}\left(u,v\right)h_{j,\alpha }\left(u,v\right)+e^{-T_{j}-T_{k}}g_{j,\alpha }\left(u,v\right)h_{k,\alpha }^{*}\left(u,v\right)\right\}. \end{array}$$

**Figure 2:**
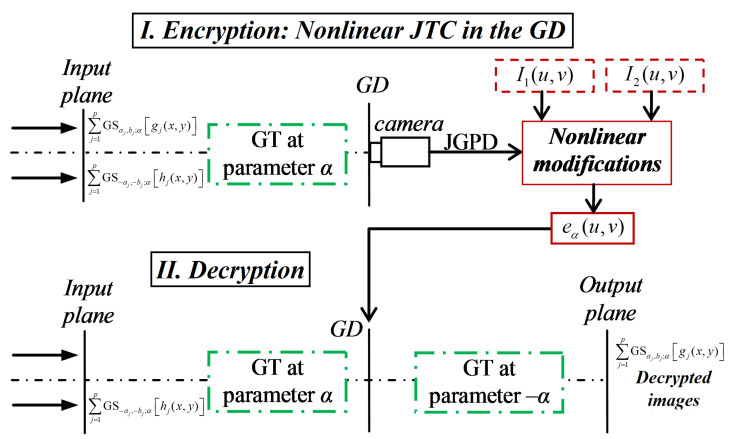
Schematic representation of the optical setup. The encryption scheme is based on a JTC in the GD and the decryption scheme is composed of two successive GTs.

The encrypted image $e{}_{\alpha }\left(u,v\right)$ is a real-valued distribution that has $2p^{2}$ terms and it is computed from the following three intensities: $\mathrm{JGPD}{}_{\alpha }\left(u,v\right)$, $I_{1}\left(u,v\right)$ and $I_{2}\left(u,v\right)$. The two general terms of the double summation in Equation (6) are noisy data distributions that represent the DRPE in the GD for all the original images to encrypt along with the 2p RPMs, $r_{j}\left(x,y\right)$ and $h_{j}\left(x,y\right)$. Figure 2 shows the optical encryption scheme (part I) based on a fully phase nonzero-order JTC architecture in the GD and the optical decryption scheme (part II) based on two successive GTs. The security keys of the encryption scheme are the 2p RPMs $r_{j}\left(x,y\right)$ and $h_{j}\left(x,y\right)$ and the rotation angle of the GT operator. The RPM $r_{j}\left(x,y\right)$ is used to spread the information content of each grayscale image $f_{j}\left(x,y\right)$ encoded in phase onto the encrypted distribution $e{}_{\alpha }\left(u,v\right)$.

### 2.2. Decryption Scheme

In the decryption scheme (Figure 2, part II), the *p* RPMs $h_{l}\left(x,y\right)$ are placed at the input plane of the decryption scheme by using the generalized shift operators ${\mathrm{GS}}_{-a_{l},-b_{l};\alpha }$. Then, the encrypted image $e_{\alpha }\left(u,v\right)$ located in the GD is multiplied by the GT of the input plane of the decryption scheme and the result is

(7) $$\begin{array}{cc} d{}_{\alpha }\left(u,v\right)= & e{}_{\alpha }\left(u,v\right)\sum_{l=1}^{p}G^{\alpha }\left\{{\mathrm{GS}}_{-a_{l},-b_{l};\alpha }\left[h_{l}\left(x,y\right)\right]\right\}=e{}_{\alpha }\left(u,v\right)\sum_{l=1}^{p}e^{T_{l}}h_{l,\alpha }\left(u,v\right)\\ = & \frac{1}{I_{2}\left(u,v\right)}\sum_{j=1}^{p}\sum_{k=1}^{p}\sum_{l=1}^{p}\{e^{T_{j}+T_{k}+T_{l}}g_{k,\alpha }^{*}\left(u,v\right)h_{j,\alpha }\left(u,v\right)h_{l,\alpha }\left(u,v\right)\\ & +e^{-T_{j}-T_{k}+T_{l}}g_{j,\alpha }\left(u,v\right)h_{k,\alpha }^{*}\left(u,v\right)h_{l,\alpha }\left(u,v\right)\}, \end{array}$$

where $T_{l}=i2\pi \left(b_{l}u+a_{l}v\right)\csc\alpha$ and this equation has $2p^{3}$ terms. The two general terms of the triple summation in Equation (7) are composed of the multiplication of different pure linear phase terms and the products between the data distributions $g_{k,\alpha }^{*}\left(u,v\right)$, $h_{j,\alpha }\left(u,v\right)$ and $h_{l,\alpha }\left(u,v\right)$, and $g_{j,\alpha }\left(u,v\right)$, $h_{k,\alpha }^{*}\left(u,v\right)$ and $h_{l,\alpha }\left(u,v\right)$, respectively. Each general term of this triple summation is divided by the nonlinear term $I_{2}\left(u,v\right)$. The first general term of Equation (7) corresponds to different noisy data distributions at the output plane of the decryption system, and the second general term of Equation (7) allows the separation of the data distribution $g_{j,\alpha }\left(u,v\right)$ from the product given by the multiplication of the data distributions $g_{j,\alpha }\left(u,v\right)$, $h_{k,\alpha }^{*}\left(u,v\right)$ and $h_{l,\alpha }\left(u,v\right)$, when l=j. The output plane of the decryption scheme is given by the GT at parameter −α of Equation (7). The resulting output plane has several distributions spatially separated. The second term of the triple sum on the right side of Equation (7) retains the most relevant information in order to retrieve the *p* grayscale images that were encrypted. The *p* decrypted grayscale images are centered at coordinates $\left(a_{j},b_{j}\right)$ and the other distributions in the output plane of the decryption scheme are spatially separated distributions from these *p* decrypted grayscale images. Therefore, the GT at parameter −α of the second term of the triple sum on the right side of Equation (7) is

(8) $$\begin{array}{cc} \hat{d}\left(x,y\right)= & G^{-\alpha }\left\{\frac{\left[\sum_{j=1}^{p}e^{-T_{j}}g_{j,\alpha }\left(u,v\right)\right]\left[\sum_{l=1}^{p}\sum_{k=1}^{p}e^{T_{l}-T_{k}}h_{l,\alpha }\left(u,v\right)h_{k,\alpha }^{*}\left(u,v\right)\right]}{\sum_{j=1}^{p}\sum_{k=1}^{p}e^{T_{j}-T_{k}}h_{j,\alpha }\left(u,v\right)h_{k,\alpha }^{*}\left(u,v\right)}\right\}\\ = & \sum_{j=1}^{p}{\mathrm{GS}}_{a_{j},b_{j};\alpha }\left[g_{j}\left(x,y\right)\right], \end{array}$$

the result of this equation is obtained when $-a_{l}=-a_{j}$, $-b_{l}=-b_{j}$ and l=j. Each term of the Equation (8) is multiplied by a linear phase term and the complex conjugate of $r_{j}\left(x-a_{j},y-b_{j}\right)$ in order to obtain a version of the decrypted grayscale image ${\hat{f}}_{j}\left(x,y\right)$ at coordinate $\left(a_{j},b_{j}\right)$ given by

(9) $$\begin{array}{cc} 2\pi {\hat{f}}_{j}\left(x-a_{j},y-b_{j}\right)= & \arg\{\exp\left\{i2\pi \left[b_{j}\left(x-\frac{a_{j}}{2}\right)+a_{j}\left(y-\frac{b_{j}}{2}\right)\right]\cot\alpha \right\}\\ & \times {\mathrm{GS}}_{a_{j},b_{j};\alpha }\left[g_{j}\left(x,y\right)\right]r_{j}^{*}\left(x-a_{j},y-b_{j}\right)\}, \end{array}$$

where arg is the phase of a complex-valued function. If the *p* keys RPMs $h_{j}\left(x,y\right)$ and the key RPM $r_{j}\left(x,y\right)$ applied in the decryption scheme are the same keys used in the encryption system, the decrypted grayscale image ${\hat{f}}_{j}\left(x,y\right)$ is a replica of the grayscale image $f_{j}\left(x,y\right)$ that was encrypted. The nonlinear modifications of the JGPD at the output plane of the encryption scheme allow obtaining a correct retrieval of the original grayscale image in the decryption scheme.

## 3. Numerical Simulations

**Figure 3:**
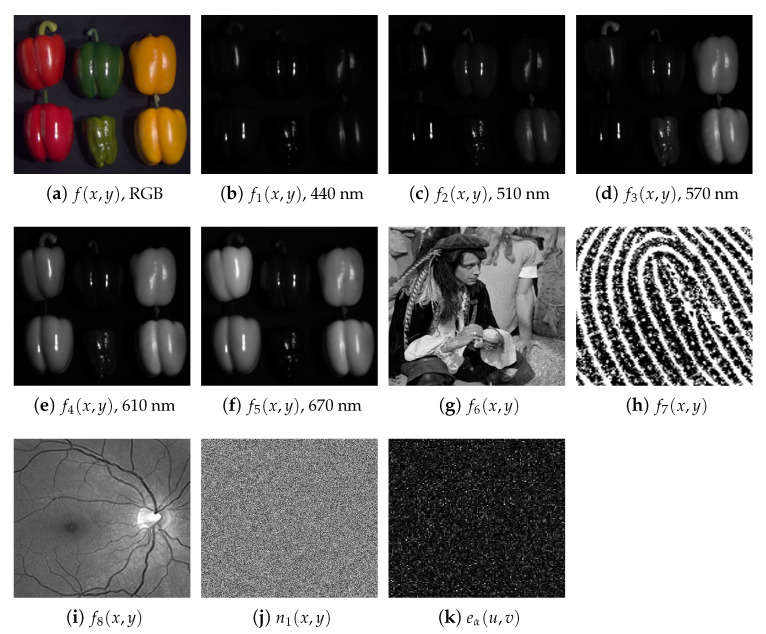
(**a**) Original image f(x,y) in the RGB color space. Multispectral image to encrypt corresponding to f(x,y) with five different color (wavelength, λ) channels: (**b**) $f_{1}\left(x,y\right)$ at λ=440 nm, (**c**) $f_{2}\left(x,y\right)$ at λ=510 nm, (**d**) $f_{3}\left(x,y\right)$ at λ=570 nm, (**e**) $f_{4}\left(x,y\right)$ at λ=610 nm and (**f**) $f_{5}\left(x,y\right)$ at λ=670 nm. Color image f(x,y) and its multispectral components were obtained from [48]. The remaining original grayscale images to encrypt: (**g**) $f_{6}\left(x,y\right)$, (**h**) $f_{7}\left(x,y\right)$ and (**i**) $f_{8}\left(x,y\right)$. (**j**) Image of the random distribution $n_{1}\left(x,y\right)$ of the RPM $h_{1}\left(x,y\right)$. (**k**) Encrypted image $e{}_{\alpha }\left(u,v\right)$ for the rotation angle α=0.775π.

In this section, we compute the numerical simulations of the encryption and decryption schemes for multiple images presented in Section 2. The resolution of the grayscale images used in these numerical simulations is 512×512 pixels (M=N=512). The images in Figure 3 show the results for the encryption scheme described in Section 2.1. We have selected eight original grayscale images to encrypt (j=1,2,3,…,8 and p=8). These images have different details and frequency spectra, and they could be used for different purposes. The first five grayscale images correspond to a multispectral image of peppers; each color component of this multispectral image is processed as a grayscale image. The original image related to the multispectral image of peppers in the RGB color space is shown in Figure 3a, and the five grayscale images $f_{j}\left(x,y\right)$ at j=1,2,3,4,5 taken from the multispectral image are depicted in Figure 3b–f. These first five grayscale images were taken from [48]. The five color (wavelength, λ) channels captured for the multispectral image of peppers are $f_{1}\left(x,y\right)$ at λ=440 nm, $f_{2}\left(x,y\right)$ at λ=510 nm, $f_{3}\left(x,y\right)$ at λ=570 nm, $f_{4}\left(x,y\right)$ at λ=610 nm and $f_{5}\left(x,y\right)$ at λ=670 nm. The remaining three original grayscale images to encrypt correspond to the photo of a person and two biometric signals given by the images of a fingerprint and a retina. These last three original grayscale images are displayed in Figure 3g–i. The image for the random code $n_{1}\left(x,y\right)$ of the RPM $h_{1}\left(x,y\right)$ is presented in Figure 3j. The random codes $s_{j}\left(x,y\right)$ of the RPMs $r_{j}\left(x,y\right)$ and $n_{j}\left(x,y\right)$ of the RPM $h_{j}\left(x,y\right)$ with j≠1 have different values but a similar appearance to the random code $n_{1}\left(x,y\right)$. The encrypted image $e{}_{\alpha }\left(u,v\right)$ for the rotation angle α=0.775π is shown in Figure 3k, which is a noisy distribution that does not reveal any information of the original images $f_{j}\left(x,y\right)$. The security keys of the encryption scheme are represented by the sixteen RPMs ($r_{j}\left(x,y\right)$ and $h_{j}\left(x,y\right)$) and the rotation angle of the GT operator.

**Figure 4:**
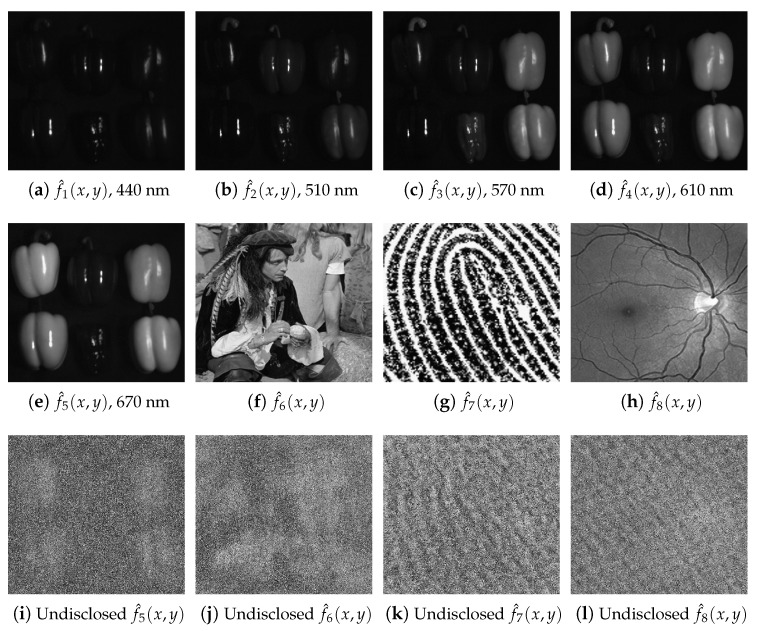
(**a**–**h**) Correct decrypted images ${\hat{f}}_{j}\left(x,y\right)$ at j=1,2,3,…,8, when the correct seventeen security keys RPMs, given by the sixteen RPMs ($r_{j}\left(x,y\right)$ and $h_{j}\left(x,y\right)$) and the rotation angle of the GT operator, are used. (**i**–**l**) Wrong decrypted images ${\hat{f}}_{5}\left(x,y\right)$, ${\hat{f}}_{6}\left(x,y\right)$, ${\hat{f}}_{7}\left(x,y\right)$ and ${\hat{f}}_{8}\left(x,y\right)$, respectively, when the incorrect security key RPM $h_{7}\left(x,y\right)$ and the other correct sixteen security keys (the remaining fifteen RPMs and the rotation angle of the GT operator) are used in the decryption scheme.

Initially for the decryption computation, we use the same values of the security keys given by the sixteen RPMs ($r_{j}\left(x,y\right)$ and $h_{j}\left(x,y\right)$) and the rotation angle of the GT operator, which were used in the encryption computation. Therefore, the right decrypted images ${\hat{f}}_{j}\left(x,y\right)$, which are replicas of the original images $f_{j}\left(x,y\right)$, are displayed in Figure 4a–h. If a wrong security key, for instance, the RPM $h_{7}\left(x,y\right)$, and the other sixteen correct security keys (the remaining fifteen RPMs and the rotation angle of the GT operator) are used in the decryption scheme, we obtain the noisy decrypted images depicted in Figure 4i–l. For the last numerical simulation, the decrypted images ${\hat{f}}_{j}\left(x,y\right)$ with j=1,2,3,4 are also noisy random distributions with a similar appearance to the decrypted image shown in Figure 4i. When another security key different from the RPM $h_{7}\left(x,y\right)$ is wrong for the decryption computation, we will also obtain noisy decrypted images very similar to the distributions displayed in Figure 4i–l. If the new nonlinear terms $I_{1}\left(u,v\right)$ and/or $I_{2}\left(u,v\right)$ defined in Equation (5) were not applied to compute the encrypted image of Equation (6) or the definitions of these two nonlinear terms were different from those in Equation (5), we would obtain multiple noisy decrypted images very alike to the images depicted in Figure 4i–l. Thus, the correct simultaneous retrieval of the original images at the output plane of the decryption scheme is only possible when all the security keys have the same values for the encryption and decryption computations, and the two nonlinear terms $I_{1}\left(u,v\right)$ and $I_{2}\left(u,v\right)$ of Equation (5) are applied in the definition of the encrypted image given by Equation (6).

The most used metric to evaluate the quality of the decrypted images is the root mean square error (RMSE), which is defined by [15]

(10) $$\mathrm{RMSE}={\left(\frac{\sum_{x=1}^{M}\sum_{y=1}^{N}{\left[f\left(x,y\right)-\hat{f}\left(x,y\right)\right]}^{2}}{\sum_{x=1}^{M}\sum_{y=1}^{N}{\left[f\left(x,y\right)\right]}^{2}}\right)}^{\frac{1}{2}}.$$

On the one hand, the RMSE values close or equal to 0 correspond to decrypted images very similar to the original ones, thus indicating a good image quality for the retrieved signal. On the other hand, the RMSE values close to 1 usually correspond to noisy decrypted signals that do not resemble the original images. The RMSEs between the original images $f_{j}\left(x,y\right)$ of Figure 3b–i, and the right decrypted images of Figure 4a–h are values between 0.015 and 0.047. Finally, the RMSEs between the original images $f_{j}\left(x,y\right)$ of Figure 3f–i and the undisclosed decrypted images of Figure 4i–l are values between 0.817 and 0.924.

Several possible drawbacks of the proposed multiple-image encryption–decryption scheme could arise when the total number *p* of images to encrypt is quite large, assuming that the resolution of each image remains unchanged. For example, in its optoelectronic implementation, the possibility to place a large number of images for encryption in the input plane of the JTC may be limited by the resolution of the display used in the input plane (e.g., a phase-only spatial light modulator). The computation time for the numerical simulations of the proposed encryption system will increase as the total number *p* of the original images to be encrypted increases, because the digital images needed to compute the three intensities ($\mathrm{JGPD}{}_{\alpha }\left(u,v\right)$, $I_{1}\left(u,v\right)$ and $I_{2}\left(u,v\right)$) of the encrypted image will have a bigger resolution. Finally, the management and distribution of the security keys of the proposed encryption–decryption scheme can be a bit more complicated due to the large number of security keys given by the 2p RPMs.

We consider the key space of the proposed encryption and decryption schemes as all possible combinations of the seventeen security keys given by the sixteen RPMs ($r_{j}\left(x,y\right)$ and $h_{j}\left(x,y\right)$) and the rotation angle of the GT operator. In reference [26], it was found that the sensitivity on the values of the rotation of the GT operator in order to retrieve a good quality decrypted image was of the order of $4\times 10^{7}$. All the sixteen RPMs are images with a resolution of 512×512 pixels and the possible values of these pixels are 256 different values. Therefore, the number of possible combinations of the sixteen RPMs is of the order of $256^{\left(16)(512)(512\right)}=256^{4194304}$ [29]. Finally, the total key space of the proposed encryption and decryption schemes is given by the product of the sensitivity on the values of the rotation of the GT operator and the number of possible combinations of the sixteen RPMs: $\left(4\times 10^{7}\right)\left(256^{4194304}\right)$. This total key space is very large, and a brute force attack for the encryption and decryption schemes of this work would be impractical. An improvement in the security of the proposed encryption scheme against CPA, KPA and COA is obtained due to the nonlinear modifications applied in this work; such nonlinear modifications are the phase encoding of the original images and the operations performed on the JGPD in order to obtain the encrypted image. This fact was shown in references [15,16,17,18,19,22,23,26,27].

## 4. Conclusions

A new encryption–decryption scheme was proposed for multiple images based on a nonlinear fully phase JTC architecture in the GD. The proposed security system can encrypt multispectral or color images considering each color channel as a grayscale image. The encryption system can also protect binary and biometric images. The proposal incorporates two nonlinear modifications: the phase encoding of the original grayscale images to encrypt and the nonlinear operations introduced in the JGPD. The retrieval of multiple images due to the nonlinear modifications provides a highly secure encryption–decryption system, which achieves an excellent quality of the decrypted images. The security keys of the proposed encryption and decryption schemes are represented by the RPMs $r_{j}\left(x,y\right)$ and $h_{j}\left(x,y\right)$ and the rotation angle of the GT operator. Only the correct values of the security keys permit a proper simultaneous retrieval of the original images in the decryption scheme. The proposed simultaneous encryption scheme has a fast computation time in comparison to previous proposals that sequentially encrypt multiple grayscale images. In our proposal, the encryption of up to eight images is obtained by simultaneously displaying the whole set of signals in the input plane of the encryption stage. In addition, the final encrypted distribution is nonlinearly computed by using only three intensity distributions. The proposed encryption–decryption scheme is more secure against several plaintext attacks because of the phase encoding of the original images to encrypt, the nonlinear operations applied over the JGPD and the larger key space of the proposed encryption scheme.

## Data Availability

The supporting information can be found from the corresponding author.

## Abbreviations

The following abbreviations are used in this manuscript:

CPA	Chosen-Plaintext Attack
COA	Ciphertext-Only Attack
DRPE	Double Random-Phase Encoding
FrFD	Fractional Fourier Domain
GD	Gyrator Domain
GT	Gyrator Transform
KPA	Known-Plaintext Attack
JGPD	Joint Gyrator Power Distribution
JTC	Joint Transform Correlator
RPMs	Random-Phase Masks
RMSE	Root Mean Square Error

## Appendix A. The Gyrator Transform Operator

The Gyrator transform (GT) operator is a linear integral transform that maps a two-dimensional function f(x,y) onto function $f_{\alpha }\left(u,v\right)$, where the parameter α is the rotation angle. The GT operator at parameter α is defined by [49]

(A1) $$\begin{array}{c} f_{\alpha }\left(u,v\right)=G^{\alpha }\left\{f\left(x,y\right)\right\}=\int_{-\infty }^{+\infty }\int_{-\infty }^{+\infty }f\left(x,y\right)K_{\alpha }\left(u,v,x,y\right)dxdy, \end{array}$$

(A2) $$\begin{array}{cc} \; & K_{\alpha }\left(u,v,x,y\right)=C_{\alpha }\exp\left\{i2\pi \left[(uv+xy)\cot\alpha -(vx+uy)\csc\alpha \right]\right\},\;C_{\alpha }=\frac{1}{\left|\sin\alpha \right|}, \end{array}$$

where $K_{\alpha }$ is the kernel of the GT operator, the values of the rotation angles are in the interval of 0≤α<2π, the variables *x* and *y* represent the coordinates at the spatial domain and the variables *u* and *v* denote the output coordinates in the Gyrator domain (GD). When α=0, the GT operator reduces to the identity transform. When α=π/2, the GT operator corresponds to the direct Fourier transform with rotation of the coordinate at π/2. When α=π, the reverse transform is derived from the GT operator. When α=3π/2, the GT operator is the inverse Fourier transform with rotation of the coordinate at π/2 [49]. The inverse GT operator corresponds to the GT operator at rotation angle −α. The GT operator is additive with respect to the rotation angle, $G^{\alpha }G^{\beta }=G^{\alpha +\beta }$.

The kernel of the GT operator is the product of the hyperbolic and plane waves, and the kernel of the fractional Fourier transform operator is the product of the spherical and plane waves [26]. The properties of the GT operator and the fractional Fourier transform operator have some aspects in common, because these operators are special cases of the linear canonical transforms [27].

The optical GT operator was implemented in [50,51] by means of a setup that uses three generalized lenses with a fixed distance between them. Each generalized lens is composed of two thin cylinder lenses and the rotation of these cylindrical lenses control the value of the rotation angle α.

## Appendix B. Generalized Shift Operator

We use the definition of the generalized shift operator proposed in [52], which is a simultaneous application of a spatial shift and a modulation by a pure linear phase term over a function f(x,y). The generalized shift operator at parameters $x_{0}$, $y_{0}$ and α is defined by

(A3) $$\begin{array}{c} {\mathrm{GS}}_{x_{0},y_{0};\alpha }f\left(x,y\right)=\exp\left\{-i2\pi \left[y_{0}\left(x-\frac{x_{0}}{2}\right)+x_{0}\left(y-\frac{y_{0}}{2}\right)\right]\cot\alpha \right\}f\left(x-x_{0},y-y_{0}\right). \end{array}$$

The generalized shift operator forms a commutative group for the rotation angle α. The composition law is ${\mathrm{GS}}_{x_{1},y_{1};\alpha }{\mathrm{GS}}_{x_{2},y_{2};\alpha }={\mathrm{GS}}_{x_{1}+x_{2},y_{1}+y_{2};\alpha }$. The usual traslation operator is obtained from the generalized shift operator when the rotation angle is α=π/2; hence, ${\mathrm{GS}}_{x_{0},y_{0};\pi /2}f\left(x,y\right)=f\left(x-x_{0},y-y_{0}\right)$. The GT operator with the rotation angle α of the generalized shift operator presented in Equation (A3) is

(A4) $$\begin{array}{c} G^{\alpha }\left\{{\mathrm{GS}}_{x_{0},y_{0};\alpha }f\left(x,y\right)\right\}=\exp\left\{-i2\pi \left(y_{0}u+x_{0}v\right)\csc\alpha \right\}f_{\alpha }\left(u,v\right). \end{array}$$

The generalized shift operator does not introduce a shift over the result of the GT operator. This property is very useful for centered optical systems [52].
